# Stress and substance use among sexual and gender minority individuals across the lifespan

**DOI:** 10.1016/j.ynstr.2018.100146

**Published:** 2018-12-27

**Authors:** Mike C. Parent, Andrew S. Arriaga, Teresa Gobble, Lexie Wille

**Affiliations:** University of Texas at Austin, Department of Educational Psychology, Sanchez 262H, Austin, TX, 78749, USA

**Keywords:** Sexual minorities, Gender minorities, LGBT, Substance use, Health disparities, Minority stress

## Abstract

Sexual and gender minority (SGM) individuals face marked disparities in substance use. The present narrative review explores research on substance use in SGM communities using a minority stress theory lens. We define the SGM population and minority stress, and explore stresses and substance use disparities in adolescence, adulthood, and older age. Though research on this topic is beginning to highlight the relationship between stress and substance use for SGM individuals, more work is needed on older SGM populations and in translating research findings to effective interventions.

## 1 Introduction

Disparities in substance use rates exist for sexual and gender minority (SGM) individuals across the lifespan (Saewyc, 2011). Research on SGM population health has long abandoned views that SGM identities or related behaviors are themselves pathological, in favor of contextual understanding of the unique stresses faced by SGM individuals (Meyer, 1995, 2003). Among the theoretical frameworks used to understand health disparities among SGM populations, minority stress theory (Meyer, 1995, 2003) is the current dominant theoretical paradigm. This framework emphasizes both social/environmental and internal stressors in influencing minority health disparities, including substance use (Goldbach et al., 2014). The present review will use minority stress theory as an organizing framework to understand health disparities among SGM populations across the lifespan.

## 2 Sexual and gender minorities

Sexual minority individuals are persons whose sexual identity or behaviors are not exclusively heterosexual. Although the term lesbian, gay, and bisexual (LGB) has often been used to refer to such individuals, this three-class category has been recognized to be insufficient to capture the natural variation in human sexual identity or expression (Moradi et al., 2009). Examples of sexual minority identities that fall outside of this triad of terms includes identities such as omnisexual or pansexual (sometimes used to avoid the implication of gender as a binary perceived to be implicit in the term “bisexual,” or to make clear the potential openness to partners who are transgender or other gender identities), queer, fluid, two-spirited, or other identities (Miller et al., 2016; Robinson, 2017).

Gender minority individuals are those for whom sex assigned at birth (i.e., the sex listed on a birth certificate) does not align with gender identity (American Psychological Association, 2015). The terms transgender, or transgender and gender nonconforming (TGNC), are often used as umbrella terms to include a range of individuals who may be transgender (whether or not they have undergone any aspects of social, hormonal, or surgical transition); the term “cisgender” applies to individuals whose sex assigned at birth aligns with gender identity. In addition, some individuals may identify as gender non-binary, or agender (American Psychological Association, 2015), identities that reflect that traditional gender identity labels may not apply to the indivdual. For example, an individual may be assigned female at birth and undertake hormonal transition to masculinize appearance, but may not identify as a man—thus, the traditional term “female-to-male transgender” would apply in the sense of the medical procedures undertaken, but not in terms of the identity of the individual. Although a heterogenous population, TGNC persons share many experiences that have led researchers to often examine TGNC persons as a group (Hendricks and Testa, 2012). The proliferation of labels used to describe sexual and gender identities may reflect greater visibility and social engagement in SGM communities, as well as online resources that facilitate easier communication among this numerically small population (Kuper et al., 2012).

It is important to note that the collective term SGM is intended to highlight the many shared experiences of sexual minorities and gender minorities. Indeed, research supports that many of the same processes underlie marginalization and victimization of individuals across the SGM continuum (Meyer, 2003). However, the term is not intended to be used to treat all individuals who fall within the SGM umbrella as though they shared precisely the same experiences (Worthen, 2013). Indeed, important variations exist between individuals in the categories (e.g., lesbian women, gay men, and transgender men have many similarities and differences), and between individuals within the categories. For example, the experiences of transgender individuals who reliably “pass” as their gender identification versus those who do not reliably pass. Similarly, bisexual individuals who are in same-gender relationships may be perceived by others to be gay or lesbian, and those who are in other-gender relationships may be perceived by others to be heterosexual. Further intersections exist among intersections of identities such as race/ethnicity or socioeconomic status (Begun and Katteri, 2016). Thus, there are many cases in which grouping SGM populations together may be reasonable, and other instances in which it is vital to understand specific groups’ experiences (Worthen, 2013).

## 3 Minority stress theory framework

Minority stress theory (Meyer, 1995, 2003) is the leading paradigm in understanding health disparities among SGM populations. Minority stress theory postulates that minority individuals experience stressors over and above the stresses experienced by non-minority persons. Minority stressors are characterized by being (a) unique, in that they are specifically directed at minority groups; (b) chronic, in that they are recurrent or pervasive rather than one-time events; and (c) socially-based, in that they are founded in social convention or ideology rather than rational grounds. For example, an individual who is a SGM may hear anti-gay slurs being used often in a workplace. Such an event is unique (the individual does not hear “heterosexual” or “straight” used as slurs), chronic (the slurs are used as part of a common vernacular rather than being a one-time occurrence), and socially-based (in that one can infer that if a word for a group is used as an expletive, it is because that group is viewed as being socially devalued).

Further, minority stress theory posits other variables that may influence the relation between stressors and negative outcomes, including substance use. Among these, the most examined in research has been coping and social support. While adaptive coping has been demonstrated to be a protective factor for SGM individuals, research on social support and community involvement has been mixed. This may be due, in part, to social support sometimes being operationally defined as involvement with the SGM communities. Pursuant to minority stress theory as applied to SGM persons, we will first explore stressors and coping that may manifest for SGM persons.

## 4 Minority stress

SGM individuals do, of course, encounter the same stressors as heterosexual/cisgender individuals. As such, many of the same substance-use related stressors would apply to SGM populations, such as the presence of mood disorders, trauma histories, poor emotional regulation, or interpersonal stresses (Aldao et al., 2010; Jacobsen et al., 2001; Kotov et al., 2010). Beyond the stresses that most individuals experience, SGM individuals may also experience minority stress. As mentioned, minority stressors are those which are *unique*, *chronic*, and *socially-constructed*. Distal events are events in the environment, and have been conceptualized as including both political/social policy (such as the presence of absence of gender- and sexual orientation-based anti-discrimination laws, state laws that prohibited same-sex marriages [in the United States, prior to the passage of marriage equality nationally]) and social/interpersonal stressors (such as harassment, discrimination, and violence; Meyer, 2003).

In addition to distal stressors, SGM individuals may encounter proximal minority stressors. Proximal minority stressors are those which occur internally to an individual as a function of external stressors. Examples include concealment of sexual orientation (driven from a need or perceived need to hide one's sexual orientation and/or gender identity for safety or security reasons), internalized homo/bi/transphobia (internalization of negative social messages and adoption of these messages into cognitive schema about oneself and others), and expectations of rejection (entering into interactions with others with the anticipation of negativity, and so eliciting negative reactions through being guarded or confrontational).

Minority stress experiences exert a cost to the physical capacity to handle stress. Minority stressors have been linked to increased cortisol levels (Huebner and Davis, 2005) and immune system and autonomic nervous system dysregulation (Lick et al., 2013). Minority stressors may also facilitate the development of post-traumatic stress disorder (PTSD). Such an association may occur because experiences of minority stress may include life-threatening events, such as being assaulted (Friedman et al., 2011). In addition, non-life-threating but pervasive stressors may promote the development of PTSD-like symptoms (Alessi et al., 2013; Roberts et al., 2010), and substance use may be used as an attempt to cope with the symptoms of PTSD (Jacobsen et al., 2001).

## 5 Neurobiology of chronic stress

The activation of the autonomic nervous system and the hypothalamo-pituitary-adrenal axis is the primary feature of stress as defined from a neurological standpoint (McEwan, 2007). Such activation is associated with the “fight or flight” response, and is adaptive to handling immediate threats and allowing an organism to survive acute threats (McEwan, 2007). Humans, however, appear capable of levels and manifestations of worry that can result in the prolonged activation of this basic physiological response. Prolonged stress can result in elevations in blood pressure, elevations in heart rate, buildup of cortisol, and other physiological processes that may result in or increase the risk for conditions such as heart disease, cardiovascular illness, weakening of the immune system, and other pathologies. Research has supported that stress, and even the anticipation of stressful events, can result in negative physiological changes in individuals even when the chronic events are not life-threatening (Chandola et al., 2006; Miller et al., 2007; Reiche et al., 2004).

Regarding substance use, animal models have demonstrated that various types of stressors are associated with self-administration of substances including opiates, stimulants, nicotine and alcohol (Lu and Shaham, 2005). The link between stress and substance use is believed to be related to the activation of pathways and brain centers related to rewards, including the mesolimbic dopaminergic pathways and the ventral striatum (e.g., Pierce and Kumaresan, 2006; Oswald et al., 2005). In humans, this pathway involves not only direct experience with stress but also the anticipation of aversive stimuli (e.g., Jenson, 2003). Thus, these neurological processes tie in directly with both proximal and distal stressors in the minority stress model to facilitate maladaptive coping including substance use.

## 6 Coping and social support

Coping and social support are important aspects of the minority stress model. Although research on SGM social support has demonstrated that it may be a protective factor against minority stressors or substance use (e.g., Lehavot and Simoni, 2011; Pflum et al., 2015), many assessments of SGM community involvement do not delineate the types of activities one engages in. Two individuals might rate themselves as highly active in SGM communities—one because they are actively involved in organizing civil rights events, legal actions related to discrimination, or initiatives to promote the public health of SGM persons, and the other because they are highly involved in nightclub or circuit party events at which drug use is very common (Halkitis and Parsons, 2002; Theodore et al., 2014). Thus, assessing community involvement as social support does not appropriately tease apart the type of involvement, and research on SGM community involvement has found inconsistent relationships between community involvement and substance use and abuse among SGM persons. More work is needed to directly examine emotional and instrumental support, as well as the utility of shared activism in promoting mental health, among SGM populations.

## 7 Minority stress and substance use among SGM persons throughout the lifespan

The minority stress model has been explored within myriad contexts. We will examine stress and substance use disparities across the lifespan, focusing on three groups: adolescents and youth (focusing on individuals under 18) and emerging adults and adults (those over 18). We will also explore disparities in substance use among older SGM adults (though, studies on older adults vary widely in what is considered “older”).

## 8 Adolescence

It is during adolescence that many TGNC individuals often begin to recognize their same-sex attraction and/or TGNC gender identity (Bockting and Coleman, 2007; Floyd and Bakeman, 2006). The recognition of same-sex attraction and TGNC gender identity, and engagement in sexual activity may or may not coincide with “coming out” experiences (i.e., disclosing one's SGM identity to others). However, regardless of disclosure of identities, youth with SGM identities face minority stressors and may be at risk for substance use. The experience of chronic stress in adolescence is associated with prolonged damage to hippocampal development in animal models (McEwen, 2007); extrapolated to humans, these disparities in the treatment of SGM youth may set a course in adolescence for later adult health disparities.

## 9 Stressors and substance use

Youth with SGM identities face several potential minority stressors. Experiences of bullying and harassment in schools have been a focus of heavy investigation. Youth with SGM identities face markedly elevated rates of bullying in schools from peers (Collier et al., 2013). Evidence also indicates that these experiences are often not reported to school officials and that when they are, they are often ignored or handled poorly (Kosciw et al., 2016).

Importantly, though being targeted for harassment is common, it is not necessary for SGM individuals to be the specific target of anti-SGM attitudes in schools for minority stress processes to activate. Homophobic and transphobic insults are common among youth, particularly boys, and are often applied to individuals who are not known or even suspected of actually being SGM (Birkett and Espelage, 2015; Collier et al., 2012; Espelage et al., 2008). Thus, a pervasive culture of apparent anti-SGM sentiment would make a school environment feel unsafe or dangerous for many SGM youth. In such an environment, these distal stressors may promote proximal stressors (e.g., concealment of sexual orientation or gender identity, internalization of the negative messages). Research has reliably linked both distal and proximal minority stressors to elevated substance use rates among SGM youth (Goldbach et al., 2014).

In the United States, many individuals initiate use of some substances during adolescence (Kann et al., 2016). However, youth with SGM identities demonstrate disparities in substance use across multiple substances (Marshal et al., 2008). Data from the youth risk behavior survey indicates that sexual minority youth have elevated risk for use of tobacco, alcohol, and other drugs (Kann et al., 2016). Because the national YRBS does not assess TGNC identities, parallel data do not exist for those youth. However, other national data collection efforts have sampled TGNC youth, and have demonstrated elevated rates of substance use among these populations as well (Reisner et al., 2015a).

The principle form of minority stress assessed among youth with SGM identities is direct school bullying and harassment. Myriad studies have linked these elevations in bullying and harassment with elevations in substance use for SGM youth (Russell et al., 2011). Such experiences of bullying may be amplified by political contexts, such as lack of support for appropriate bathroom or locker room access for TGNC youth (Kosciw et al., 2016), or the institution of so-called “no promo homo” laws forbidding discussion of sexual and gender diversity in public schools (Barret and Bound, 2015). In addition to experiences of bullying, youth with SGM identities may encounter family rejection in reaction to their minority status (Saewyc et al., 2006); such experiences of violence are associated with both leaving the home in adolescence due to harassment, or being forced out of the home by caregivers (Keuroghlian et al., 2014). In either case, homelessness among youth with SGM identifies is associated with elevations in substance use beyond those reported by heterosexual and cisgender youth (Cochran et al., 2003).

## 10 Prevention and intervention

Means exist by which distal stressors may be ameliorated for youth with SGM identities. Research indicates that the presence of anti-bullying state laws that specifically protect youth with SGM identities from bullying may be effective in reducing bullying of students with SGM identities (Hatzenbuehler et al., 2015). However, at the same time, it is important that school staff are appropriately trained and empowered to handle such bullying complaints from students (Greytak and Kosciw, 2014). Training for school staff to reduce implicit or explicit bias against SGM students may be necessary, as evidence indicates that in many instances staff are complicit in, or even instigate, anti-SGM harassment (Gorski et al., 2013; Kosciw et al., 2016).

Regarding experiences of harassment or violence within a family unit, limited work has explored interventions to reduce parental homophobia. Some treatment research has suggested that attachment-based family therapy may be useful in family units in reducing suicidal ideation among adolescents with SGM identities (Diamond et al., 2012). However, this work has not yet been extended to substance use interventions. Similarly, limited work has examined extension of the minority stress model into the treatment of substance use among adolescents with SGM identities. Some frameworks exist in this regard (Craig, 2013; Craig et al., 2013), though clinical trials of interventions in this regard are lacking.

## 11 Emerging, middle, and young adulthood

As individuals transition to early adulthood, they may encounter significant life changes (e.g., post-secondary education, full-time work, financial independence, seeking long-term romantic partnerships; Arnett, 2005; Tucker et al., 2005). This adjustment period is often referred to as emerging adulthood—a period of time between 18 and 25 years old in which an individual makes a transition from adolescence to independent adulthood (Arnett, 2000).

SGM individuals are at an increased risk for smoking, alcohol, and other substance use at younger ages than their heterosexual and cisgender peers (Green and Feinstein, 2012; Greenwood and Gruskin, 2007; Hughes and Eliason, 2002). The stressors associated with life changes in emerging adulthood may further explain this elevated risk, as emerging adults with SGM identities may find it difficult to simultaneously manage internal sexual orientation- or gender-related identity stressors in a new environment and may also be further subjected to discrimination, marginalization, and ongoing social difficulties because of their identities (Meyer, 2003; Reed et al., 2010; Wong et al., 2010). It is common for substance use and abuse to be particularly accelerated within this population in response to the prominent distal and proximal stressors they face in a number of settings during this time (Colfax et al., 2005; Fish and Pasley, 2015; Talley et al., 2010).

## 12 Adjustment and family concerns

SGM persons who transition into more independent roles in emerging adulthood experience a number of adjustment-related challenges that may contribute to increased substance use. College samples of sexual minority men have higher rates of alcohol and substance use compared to heterosexual men of the same age (Hatzenbuehler et al., 2008; Kroshus and Davoren, 2016). Young sexual minority men are also more likely to report alcohol and substance use when compared with older sexual minority men (Salomon et al., 2009). Further, elevated substance use rates have been shown to persist throughout adulthood among men, and are associated with experiences of stress and comorbid with other health risks (Halkitis et al., 2015; Rosario et al., 2006).

Among sexual minority women, these patterns of high substance use are echoed, with elevations in smoking rates (Eisenberg and Wechsler, 2003) and alcohol consumption and alcohol-related problems (Green and Feinstein, 2012; Nawyn et al., 2000) among lesbian and bisexual women. Bisexual women appear to be at particularly heightened risk for substance use and abuse (Kerr et al., 2014). Within this age group, women with men and women partners have been found to be two times as likely to binge drink (Eisenberg and Wechsler, 2003) and to be two to three times as likely to use tobacco, marijuana, and other illicit substances (Brewster and Tillman, 2012) as women with only opposite-sex partners.

Supportive campus resources, personal sources of encouragement, and personality-related factors may particularly offset some of the substance use disparities prominent among lesbian and gay college students (Eisenberg and Wechsler, 2003; Eliason et al., 2011; Livingston et al., 2016). Elevated rates of smoking, alcohol, and drug use in TGNC individuals are associated with institutional discrimination (Grant et al., 2011).

In addition to the transitional stressors facing SGM emerging adults, ongoing family issues related to one's sexual orientation or gender identity may contribute to substance use and abuse. Compared to peers who did not experience family rejection, a lack of familial support has been linked to a 3.4 times greater likelihood of using illegal substances (Ryan et al., 2009), as well as to smoking and prescription drug abuse (Rosario et al., 2014), among sexual minority individuals. In the TGNC population, family rejection has also been found to be related to smoking and elevated drug and alcohol use (Klein and Golub, 2016). Of those who have experienced domestic violence by a family member, 47% reported elevated drinking and drug use as a means of coping (Grant et al., 2011). SGM individuals may experience hardship as a result of family rejection and conflict—including homelessness, which places individuals at an additionally heightened risk for marijuana use, alcohol abuse, and highly addictive substance use (Bruce et al., 2014; Choi et al., 2015; Cochran et al., 2002; Reback et al., 2007).

## 13 Discrimination and social norms

Social and relational factors are additionally influential in the experiences of stress and related substance use behaviors that may develop in early adulthood. As this time is often marked by greater exploration of one's social and community identity as a burgeoning adult, individuals can often be subjected to more open displays of discrimination and may in turn develop a sense of internalized negativity that often lead to drug and alcohol-based coping (Ilan H. Meyer, 2003; Weber, 2008). Experiences of discrimination contribute to great disparities in rates of tobacco and alcohol use (Pachankis et al., 2014; Puckett et al., 2017), illicit drug abuse (Feinstein and Dyar, 2017; Grant et al., 2010; Mereish et al., 2014; Pachankis et al., 2016), prescription drug misuse (Kecojevic et al., 2015), and alcohol and substance use disorders (Green and Feinstein, 2012; McCabe et al., 2010; Weber, 2008) disproportionately affecting SGM individuals. Moreover, acts of outright violence further contribute to health disparities; intimate partner violence has been linked to high levels of drug use among gay and bisexual men (Wong et al., 2010), sexual assault has been linked to elevated alcohol abuse among lesbian and bisexual women (Rhew et al., 2017), and acts of public violence have been found to contribute to an increase in both alcohol and drug use among TGNC individuals (Grant et al., 2011).

Specific subsets of the SGM population are at an increased risk for experiences of discrimination, harassment, and further increases in alcohol and drug use. For instance, like bisexual women, transgender women often report exceptionally high rates of substance use and drinking (Hughes and Eliason, 2002; Kerridge et al., 2017; Miller and Grollman, 2015; Rowe et al., 2015). Further, SGM individuals of color are regularly subjected to multiple forms of discrimination that further reinforce use disparities. In a sample of Latino gay, bisexual, and TGNC persons, polysubstance use reports were more than two times those of the national averages for the Latino population (Bruce et al., 2008). Further studies have reported high rates of hazardous alcohol use, methamphetamine, and crack cocaine use in Black sexual minority men (Jerome and Halkitis, 2009; Paul et al., 2014; Tobin et al., 2014), high rates of methamphetamine and marijuana use among Latino sexual minority men (Halkitis et al., 2014; Paul et al., 2014), greater levels of hazardous alcohol use among Black sexual minority women (Lewis et al., 2016), and prominent cigarette and ecstasy/MDMA use among Asian/Pacific Islander sexual minority men (Paul et al., 2014; Storholm et al., 2011). Other marginalized statuses, such as low socioeconomic status (Greenwood et al., 2001; Halkitis et al., 2014), gender nonconforming gender expression (Rosario et al., 2008), immigration status (Gilbert et al., 2014), and involvement in underground economies such as sex work or drug dealing (Grant et al., 2010) also play a great role in further contributing to substance abuse practices among SGM individuals.

Given the long marginalized status and relegation of SGM communities to the fringes of society, nightlife settings have often been safe havens for members of the population (Weightman, 1981) and are frequented by many emerging adults. However, this community characteristic has also contributed to longstanding norms regarding the use of substances as a form of socialization in the SGM communities, which still prevail today. This is evidenced in findings of heightened substance use among sexual minority men (Greenwood et al., 2001; Halkitis and Palamar, 2008; Kipke et al., 2007) and lesbian women (Heffernan, 1998) who frequent gay bars and parties. These norms have seemingly extended beyond nightlife settings, as it has also been found that living in predominately SGM-populated neighborhoods and being actively involved in SGM community activities may contribute to higher odds of drug use among gay and bisexual men (Carpiano et al., 2011) and cigarette use, drinking, and drug use in sexual minority women (Feinstein et al., 2017; Johns et al., 2013).

Substance use disparities also persist in the sexual and romantic endeavors in many SGM individuals. While some members of SGM communities use drugs and alcohol to enhance the pleasure of sexual encounters (Grov et al., 2013; Mitchell, 2015; Sugano et al., 2006), others, such as Black and Latino sexual minority men, have been reported doing so as a means of facilitating sexual comfort and exploration of one's sexual identity (Harawa et al., 2008; Mutchler et al., 2014). Use of substances during sexual encounters contributes to disparities in interpersonal violence (Bimbi et al., 2008; Kelly et al., 2011) and health concerns prevalent in SGM communities, including unsafe sex and the transmission of sexually transmitted infections (Greenwood and Gruskin, 2007; Lee et al., 2016).

## 14 Work, healthcare, and other public settings

When SGM individuals enter the workforce, they are often faced with new constellations of stressors. SGM individuals notably face heightened level of workplace discrimination, which includes mistreatment on the job, harassment, under-employment, and wrongful termination (Grant et al., 2011; Griffith and Hebl, 2002; Pizer et al., 2012; Ragins and Cornwell, 2001). Such actions of discrimination have been linked to especially substance use and abuse in subsets of the SGM population. Workplace harassment and stress have been linked to greater alcohol consumption and alcohol-related problems in sexual minority women employed on a college campus (Nawyn et al., 2000), job loss has been found to be related to smoking and substance use in a national subset of TGNC individuals (Shires and Jaffee, 2015), and methamphetamine and crack/cocaine use has been cited as a means of coping with work stress in a community sample of sexual minority men (Diaz, Heckert and Sánchez, 2005).

As SGM individuals progress in adulthood, they may also face greater community discrimination even beyond the workplace. Two notable domains where this commonly affects SGM persons are medical and mental health settings. SGM individuals report instances of mistreatment and enacted stigma in doctor's offices and other healthcare settings (Grant et al., 2010; Krieger and Sidney, 1997; Reisner et al., 2015b). TGNC communities have been subjected to a lack of competent and gender-affirmative routine, emergency, and transition-related care, and these negative experiences have been associated with higher rates of coping-based alcohol, cigarette, and substance use (Grant et al., 2011; Reisner et al., 2014). In some instances, marginalization of TGNC persons in medical settings may be institutionalized, as in the denial of some medical coverages for TGNC individuals not only for gender transition procedures but also for routine medical care in some instances (e.g., if an individual is able to transition from female to male on medical documentation but still requires a routine pap smear, or if an individual transitions legally from male to female and still requires prostate exams; Learmonth et al., in press; Stroumsa, 2014).

Reports of discrimination and prejudice against SGM individuals are also present in mental health care. Eliason and Hughes (2004) highlighted this in their investigation of the attitudes of substance treatment counselors toward SGM individuals. Almost half of the respondents reported negative or ambivalent attitudes about SGM clients—such attitudes may reinforce fears of disclosure in treatment settings (Eliason and Hughes, 2004). Indeed samples of SGM individuals have reported avoiding treatment for substance use disorders (McCabe et al., 2013; Salomon et al., 2009).

In addition to these experiences in health and wellness settings, TGNC individuals notably continue to experience levels of public discrimination and harassment in other settings. SGM adults who live outside of traditional settings, such as in prison, may also experience pervasive and deleterious discrimination and prejudice (Grant et al., 2011; Caspani, 2018).

## 15 Substance use progression beyond emerging adulthood

The behaviors that develop during emerging adulthood in SGM persons can serve to inform the patterns that follow individuals through the remainder of their young adult years and into middle age. Beyond emerging adulthood, gay and bisexual men have continued to exhibit elevated use of alcohol use (Stall et al., 2001), cigarettes (Stall et al., 1999; Tang et al., 2004), and other drugs (Cochran et al., 2004; Halkitis et al., 2007; Palamar et al., 2008; Stall et al., 2001), compared to heterosexual men.

Among lesbian and bisexual women experiencing minority stress, age is not supported as a protective factor in relation to alcohol use, alcohol-related problems, and substance use, as it is in heterosexual populations (Green and Feinstein, 2012; Lehavot and Simoni, 2011; Wilson et al., 2016). Sexual minority women approaching middle age have been found to maintain classification as high- and moderate-risk substance users, maintain higher drinking rates than heterosexual women, and report higher rates of cigarette use, compared to heterosexual women (Burgard et al., 2005; Cochran et al., 2013; Corliss et al., 2006; Fallin et al., 2015; Gruskin and Gordon, 2006).

Disparities in TGNC substance use have also been shown to persist over time. Among transgender women, heightened use of alcohol and various substances—including methamphetamine, marijuana, crack cocaine, and club drugs—remains elevated and associated with HIV risk (Reback and Fletcher, 2014; Santos et al., 2014). TGNC individuals also commonly report substance-based coping in response to various forms of discrimination and harassment they often face across a number of daily settings (Budge et al., 2013).

## 16 Minority stress and substance use in SGM older adults

Millions of SGM individuals in the United States are 65 years of age or older (Fredriksen-Goldsen et al., 2013; Herman et al., 2017). Older adults in the United States face unique stressor, and older adults with SGM identities may face unique forms of stress. Older SGM adults may be living with HIV as a result of seroconversion during the AIDS crisis, and may also face long-term health consequences of side effects of early HIV medications (Carr and Cooper, 2000; Vigouroux et al., 1999). Older persons with SGM identities may also have lost many friends and/or partners during the AIDS crisis (Klein and Fletcher, 1987). Many older SGM may not have come out until older adulthood, and may also have children from earlier other-sex marriages. Despite the so-called “myth of gay affluence” (i.e., the stereotype that SGM are generally well-off financially; Lee, 2000), older SGM face heightened risk of substance abuse, particularly alcohol, smoking, and prescription drug use, as well as stressors such as having a disability, having mental health concerns, being in poor physical health, and living alone (Fredriksen-Goldsen et al., 2013).

## 17 Health concerns

For older gay men living with HIV, lack of access to HIV treatments early in the progression of the disease, or use of early treatments with more significant side effects than modern medications, may have caused health issues that may persist into older age (Orlando et al., 2006). For older lesbian women, obesity and cardiovascular disease rates are elevated (Fredriksen-Goldsen et al., 2013). Limited research on older transgender persons suggests that they may be particularly disinclined to seek out medical care due to fears of stigma (Fredriksen-Goldsen et al., 2014). However, limited work has extended research on experiences of minority stress to substance use in this population.

## 18 Social isolation and rejection

Older SGM adults are twice as likely to report living alone as heterosexual adults of the same age (Gendron et al., 2013). Reasons cited for this difference in isolation include estrangement from family members based on orientation or gender identity and reduced numbers of adult children or grandchildren with which to interact (Erosheva et al., 2016). Bisexual men and women may face family or social network difficulties if a new partner is a different gender from a prior long-term partner (Fredriksen-Goldsen et al., 2014).

Older SGM adults may also face active discrimination from persons in continued care helping professions, such as nursing homes or other long term independent living facilities (Erdley et al., 2014). Older SGM adults with health insurance often avoid the healthcare system due to fears of being mistreated by healthcare providers (Ritter and Ueno, 2018). Finally, older SGM adults may be more likely to face mobility issues compared to their younger counterparts, and community resources geared toward older SGM individuals are scarce (Erdley et al., 2014). Older SGM adults, particularly gay and bisexual men, may struggle to meet romantic partners as dating ideals are often geared toward younger men (Ryan et al., 2010; Slevin and Linneman, 2010).

## 19 Substance abuse

Little research has examined substance use among older SGM adults (Heath et al., 2012). Older adults with HIV/AIDS face increased risk for alcohol, tobacco, non-Opioid prescription medications, marijuana, and stimulants (Edelman et al., 2014). Some evidence does suggest that disparities in alcohol use persist into older adulthood for gay and bisexual men, and lesbian women, and that disparities in smoking among gay and bisexual men also persist into older adulthood (Fredriksen-Goldsen et al., 2013). The lack of research in this area represents a major gap in needed knowledge for the near future, as the next generation of older adults may have substantial need for substance use treatment (Gfroerer et al., 2003; Wu and Blazer, 2011).

## 20 Summary

Extant work has uncovered significant health disparities in substance use for SGM individuals. These health disparities have been linked with both social/environmental and internal stressors within the minority stress theory framework. Health disparities for SGM individuals begin early and may persist throughout the lifespan.

Many opportunities exist for increased research on minority stress processes and the neurobiology of stress. Nearly all investigations of minority stress have drawn primarily on survey-based research methodology. While informative, the collaboration between social and behavioral health researchers, and researchers in fields such as endocrinology, neurology, and cardiology, could have important implications for understanding the process of minority stress as it relates to substance use. Further, such investigations have the potential to lead to the development of transdisciplinary approaches to treating health disparities among SGM populations. These approaches may address physiological, psychological, and behavioral health within the context of minority stress models and acknowledge the marginalization of SGM populations, including lack of access to care.

Given the evidence for the early impact of chronic stress on well-being, the development of transdisciplinary research and interventions is vital to aim toward younger populations, so as to mitigate the impact of minority stress before it blooms into major adult health disparities that have personal impacts on individuals as well as significant burdens on health care systems. Both basic research and applied interventions are needed among SGM youth, as well as professional training programs to facilitate improvements in care.

Though research on this topic has generated important findings, many gaps remain. Little is known about older SGM individuals, for whom substance use may be a looming public health crisis. As well, little is known about the intersections of identities and multiple marginalized identities beyond whether particular groups are at elevated risk for use or abuse or particular substances. Intersectional research frameworks (Parent et al., 2013) may be informative for explicating these links. Unfortunately, limited research has extended theoretical and empirical work on relations between minority stress and substance use to inform actual interventions with this at-risk population. Clinical trials of patient-centered care interventions directed at providing culturally sensitive, accessible care for substance use disorders are needed to bridge this gap between research and intervention.
